# Antibiotic use on paediatric inpatients in a teaching hospital in the Gambia, a retrospective study

**DOI:** 10.1186/s13756-018-0380-7

**Published:** 2018-07-16

**Authors:** Pa Saidou Chaw, Kristin Maria Schlinkmann, Heike Raupach-Rosin, André Karch, Mathias W. Pletz, Johannes Huebner, Ousman Nyan, Rafael Mikolajczyk

**Affiliations:** 1 PhD Programme, Epidemiology“Braunschweig-Hannover, Department of Epidemiology, Helmholtz Centre for Infection Research, 38124 Braunschweig, Germany; 2 Institute for Medical Epidemiology, Biometry, and Informatics (IMEBI), Medical Faculty of the Martin-Luther University Halle-Wittenberg, 06112 Halle (Saale), Germany; 3 Department of Epidemiology, Helmholtz Centre for Infection Research, 38124 Braunschweig, Germany; 4 German Center for Infection Research (DZIF), Hannover-Braunschweig site, 30625 Hannover, Germany; 5 Center for Infectious Diseases and Infection Control, Jena University Hospital, Am Klinikum 1, 07747 Jena, Germany; 6 Division of Paediatric Infectious Diseases, Dr. Von Hauner Children’s Hospital, Ludwig Maximilian University Munich, 80337 Munich, Germany; 7 Department of Medicine, School of Medicine and Allied Health Sciences, University of the Gambia, Edward Francis Small Teaching Hospital, Banjul, The Gambia; 8 Hannover Medical School, 30625 Hannover, Germany

**Keywords:** Paediatrics, Antibiotic use, Microbiology, Antibiotic resistance, Antibiotic stewardship

## Abstract

**Background:**

Antibiotics are useful but increasing resistance is a major problem. Our objectives were to assess antibiotic use and microbiology testing in hospitalized children in the Gambia.

**Methods:**

We conducted a retrospective analysis of paediatric inpatient data at The Edward Francis Small Teaching Hospital in Banjul, The Gambia. We extracted relevant data from the admission folders of all patients (aged > 28 days to 15 years) admitted in 2015 (January–December), who received at least one antibiotic for 24 h. We also reviewed the microbiology laboratory record book to obtain separate data for the bacterial isolates and resistance test results of all the paediatric inpatients during the study period.

**Results:**

Over half of the admitted patients received at least one antibiotic during admission (496/917) with a total consumption of 670.7 Days of Antibiotic Therapy/1000 Patient-Days. The clinical diagnoses included an infectious disease for 398/496, 80.2% of the patients on antibiotics, pneumonia being the most common (184/496, 37.1%). There were 51 clinically relevant bacterial isolates, *Klebsiella species* being the most common (12/51, 23.5%), mainly from urine (11/12, 91.7%). Antibiotic resistance was mainly to ampicillin (38/51, 74.5%), mainly reported as *Coliform species* 11/51, 21.6%.

**Conclusions:**

More than half of the admitted patients received antibiotics. The reported antibiotic resistance was highest to the most commonly used antibiotics such as ampicillin. Efforts to maximize definitive antibiotic indication such as microbiological testing prior to start of antibiotics should be encouraged where possible for a more rational antibiotic use.

## Background

Antibiotic resistance is a major problem especially in resource-limited countries where the burden of infectious diseases is high, with often higher resistance rates than in industrialized countries [1]. Children have higher risk of developing infectious diseases than adults [2, 3], and accurate aetiological diagnosis is often difficult due to the non-specific manifestation of infections in this age group [4, 5]. Microbiological investigations are therefore especially useful to confirm definitive indication of antibiotics and for their rational use on children [6], but this is a challenge in developing countries where limited laboratory testing is available [2]. In developing countries, shortages of drug supplies also often restrict prescribers to the available drugs [7].

Inappropriate antibiotic use is well described in developed countries but not as well studied in developing countries [8]. Local data on antibiotic consumption and resistance profile is useful in helping formulate policies and recommendations on antibiotic use both at local and regional levels [7]. Inappropriate prescription of antibiotics has been reported elsewhere in Africa such as in Ethiopia where a study reported up to 86.6% of antibiotics prescribed for the treatment of cough and diarrhoea among less than 60 months old children attending to hospitals were inappropriate [9]. Within the sub region, a study in Senegal reported prescription indication errors mainly with antibiotic and antimalarial drugs, and dosage errors mainly with antibiotics and antifungal drugs [10]. In the Gambia, over prescription of antibiotics among children less than 60 months old have been reported in the outpatient setting of health centres [11], but to our knowledge, data on appropriateness of antibiotic prescribing is lacking. In addition, antibiotic resistance patterns have been reported for *Streptococcus species* [12, 13], *Salmonella* [14], *Helicobacter pylori* [15], and for specific disease conditions such as severe malnutrition [16], within smaller health facilities and population. Microbiological test patterns for neonates treated with antibiotics have also been reported [17]. But national and international data on antimicrobial resistance patterns in the paediatric setting is still limited, thus affecting the development of evidence based policies and guidelines [7]**.** As far as we know there has been no published study examining antibiotic prescribing and microbiological testing patterns in the general paediatric inpatients in the Gambia.

Different bacteria use different mechanisms to develop antibiotic resistance as defined by Munita et al. [18], who classified antibiotic resistance into four major biochemical mechanisms as follows: a) modifications of the antimicrobial molecule (by chemical alterations of antibiotics and destruction of antibiotic molecule), b) prevention of antibiotics to reach target (by decreasing antibiotic penetration and increasing efflux), c) change or bypass of target sites (through target protection and modifying the target site), and d) resistance due to global cell adaptive processes. While the process of prevention of antibiotics to reach target by decreasing antibiotic penetration is mainly for gram-negative bacteria due to the presence of an outer membrane, classical antibiotics affected by resistance due to global cell adaptive processes are usually used for treating gram-positives (vancomycin and daptomycin). In developed countries, the dynamic spread of antibiotic resistance has led to the establishment of antibiotic stewardship (ABS) programs fostering prudent use of antibiotics [19–21]. Such programs are currently rare and more difficult to implement in developing countries due to limited resources [22]. In order to estimate the expected impact of an ABS-program, prior analysis of antibiotic prescribing behaviour is required. Therefore, the objectives of our study were to assess the antibiotic consumption, the antibiotic indication and dosage, and use of microbiological testing on paediatric inpatients at a teaching hospital in The Gambia. This would enable us to test our hypothesis that in addition to other possible factors, limited microbiology use contributes to limited definitive antibiotic indication and high antibiotic consumption in The Gambia. Our results would provide up-to-date information on current practice on antibiotic prescribing and antibiotic resistance patterns in the paediatric setting, and other countries are likely to face similar problems. Thus the findings support the need and would contribute evidence, for the establishment of national, regional, and global guidelines and policies to promote rational antibiotic use.

## Methods

### Setting

The study was a retrospective analysis of paediatric inpatient data from The Edward Francis Small Teaching Hospital in Banjul (EFSTH), The Gambia’s largest hospital referral centre. The hospital serves as the country’s main tertiary care centre receiving patients from the whole country. The team of medical doctors responsible for the management of patients include specialists, medical officers, and house officers.

### Data collection

We extracted the required data from the admission folders of patients aged > 28 days to 15 years admitted in 2015 (January–December), who received at least one antibiotic for at least 24 h, using Microsoft Access 2010. We excluded records of patients discharged against medical advice and admission folders with missing dates or loss of documents containing antibiotic or diagnoses details. Data extracted included: age, weight, height, sex, clinical diagnosis*,* antibiotic treatment (name, treatment duration, route and frequency of administration), and microbiology workup. All the included patients had at least one diagnosis at the time of admission; the diagnoses were mostly clinically based. All the diagnoses included were as documented on the patients’ records. We also obtained information on the total admissions during this period. In addition, we reviewed the microbiology laboratory record book to obtain separate data for the bacterial isolates and resistance test results of all the paediatric inpatients during the study period.

### Assessment of antibiotic consumption

Antibiotic consumption was assessed based on qualitative indicators which assess appropriateness of antibiotic use, and quantitative indicators which assess the volume or cost of antibiotics used [23]. For the qualitative assessment of antibiotic consumption, we used a World Health Organization (WHO) guideline to assess compliance to indication and dosing. Because under 5 year old children with community acquired pneumonia (CAP) have been reported to have the highest percentage of encounter with an antibiotic prescribed in an outpatient study in The Gambia [11], we used the clinical diagnoses of CAP to assess the antibiotics indicated, and the prescribed dosages for ampicillin and penicillin-G for treating the cases among children less than 5 years old, by comparing them to the dosage recommendations by the WHO for the treatment of severe pneumonia in this age group [24]. We restricted this analysis to clinical diagnoses of pneumonia but excluded the cases of pneumonia with other underlying diseases such as sickle cell disease or HIV, superimposed pneumonia, or cases of pneumonia with possible non-infectious causes such as aspiration pneumonia, as indicated on the clinical diagnoses records. We excluded anti-Tuberculosis drugs from the analysis of the antibiotics. We also used two of the common infectious disease diagnoses in children (sepsis and urinary tract infections (UTI)) [25, 26] to assess the use of microbiological culture results to guide definitive antibiotic indication.

For the quantitative assessment of antibiotic consumption for inpatients, we used the recommended Days of Antibiotic-Therapy (DoT)/1000 Patient-Days (PD) to assess the volume of antibiotics used in the paediatric inpatient setting [19], since the defined daily dose (DDD) is mainly indicated for adults [27] and poorly estimates antibiotic consumption in paediatrics [28]. We found DoT to be a good option to estimate antibiotic use density since it considers each antibiotic and the number of days it was used, therefore every antibiotic contributes independently to the DoT [28]. This provides a better estimate of the overall antibiotic volume and comparable with other settings [19, 23]. In addition, we also calculated the proportion of antibiotics used by any patient during the study period.

### Bacterial cultures and antibiotic resistance testing

To assess the bacteria isolates and resistance test results for the study period, we used the data we obtained from the laboratory records since we assumed that this data may be more complete than those in the admission folders. However, due to lack of the hospital numbers for some of the records, matching of these patients with the admission data was not feasible.

### Data analysis

We analyzed the data with Stata version 12 (StataCorp., College Station, TX, USA) using a complete case approach. We summarized the results into proportions, ratios, and medians. Where applicable, we compared children under 5 and those over 5 years of age. To make comparisons and test for associations for antibiotic use, we used Chi square test (for all admitted children) and Fisher’s exact test (for CAP, sepsis, and UTI diagnoses). We set statistical significance at ≤0.05.

## Results

### Diagnoses and antibiotic use

**Fig. 1:**
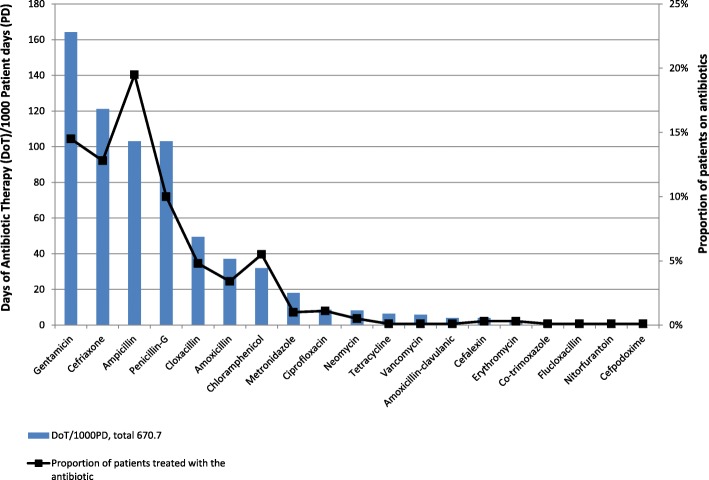
Antibiotics used: Comparing Days of Antibiotic Therapy to the proportion of patients on each antibiotic

For the year 2015 (January–December), 917 patients were admitted, 496 (54.1%) received at least one antibiotic and fulfilled the other inclusion criteria for the analysis, 181/496, 36.5% of these also received antibiotics on discharge. The total antibiotic consumption was 670.7 DoT/1000 PD. Most of the patients treated with antibiotics had at least one infectious disease diagnosis (80.2%) (Table 1), the most common were pneumonia (184/496, 37.1%) and sepsis (70/496, 14.1%). The most common antibiotics used were ampicillin (179/917, 19.5%), gentamicin (133/917, 14.5%), and ceftriaxone (117/917, 12.8%). Table 2 shows antibiotics prescribed for admitted patients during the study period, classified according to the Anatomical Therapeutic Chemical (ATC) classification system by the WHO Collaborating Centre for Drug Statistics Methodology [27]. Fig.1 compares the DoT of each antibiotic to the proportion of patients treated with each antibiotic.

**Table 1:** General characteristics of admitted patients aged > 28 days to 15 years from January–December 2015

Variable	Categories	Frequency
Total admissions		*n* = 917, %
Age	Under 5	630, 68.7%
	Over 5	287, 31.3%
Patients treated with antibiotic		*n* = 496, %
Sex	Male	302, 61.1%
	Female	192, 38.9%
Type of clinical diagnosis*	Infectious disease diagnosis	398, 80.2%
	Non-infectious disease diagnosis	300, 60.5%
	Both Infectious disease and non-infectious disease diagnosis	202, 40.7%
Age	Under 5	366, 73.8%
	Over 5	130, 26.2%
Length of hospital stay	<=7 days	264, 53.2%
	8–14 days	150, 30.2%
	> 14 days	82, 16.5%
Duration of antibiotic treatment	<=7 days	361, 72.8%
	7–14 days	100, 20.2%
	> 14 days	35, 7.1%

*not mutually exclusive, based on admission diagnosis

**Table 2:** Antibiotics used on inpatients aged > 28 days to 15 years during the period January – December 2015

Antibiotic	DoT	DoT/1000PD (Total PD = 4045)	Proportion of patients treated with the antibiotic (*n* = 917, %)	Age		
				under 5 years *n* = 630, %	5 years and more *n* = 287, %	*P* Value*
J01G aminoglycosides						
Gentamicin	664	164.2	133, 14.5%	121, 19.2%	12, 4.2%	< 0.001
Neomycin	33	8.2	5, 0.5%	5, 0.8%	0	0.154
J01D cephalosporins						
Cefriaxone	490	121.1	117, 12.8%	86, 13.7%	31, 10.8%	0.982
Cefalexin	16	4.0	3, 0.3%	3, 0.5%	0	0.231
Cefpodoxime	1	0.2	1, 0.1%	1, 0.2%	0	0.551
J01C Beta-lactam antibacterials, Penicillins						
Ampicillin	416	103.0	179, 19.5%	140, 22.2%	39, 13.6%	0.003
Penicillin-G	416	103.0	92, 10.0%	64, 10.2%	28, 9.8%	0.077
Cloxacillin	200	49.4	44, 4.8%	39, 6.2%	5, 1.7%	0.054
Amoxicillin	150	37.1	31, 3.4%	21, 3.3%	10, 3.5%	0.847
Amoxicillin-clavulanic	16	4.0	1, 0.1%	0	1, 0.3%	0.017
Flucloxacillin	6	1.4	1, 0.1%	1, 0.2%	0	0.551
J01B Amphenicols						
Chloramphenicol	129	31.9	50, 5.5%	33, 5.2%	17, 5.9%	0.078
J01XD Imidazole derivatives						
Metronidazole	73	18.0	9, 1.0%	5, 0.8%	4, 1.4%	0.209
J01 M Quinolones						
Ciprofloxacin	35	8.7	10, 1.1%	3, 0.5%	7, 2.4%	< 0.001
J01A Tetracyclines						
Tetracycline	26	6.4	1, 0.1%	0	1, 0.3%	0.093
J01XA glycopeptides						
Vancomycin	24	5.9	1, 0.1%	1, 0.2%	0	0.551
J01FA macrolides						
Erythromycin	7	1.7	3, 0.3%	2, 0.3%	1, 0.3%	0.398
J01E Sulfonamides and Trimethoprim						
Co-trimoxazole	6	1.4	1, 0.1%	1, 0.2%	0	0.551
J01XE Nitrofuran derivatives						
Nitorfurantoin	5	1.2	1, 0.1%	0	1, 0.3%	0.093
Total	2713	670.7	–	–	–	–

*Chi-squared

*DoT*: Days of Antibiotic-Therapy

*PD*: Patient-Days

### Antibiotic indication and dosage

Most of the children with CAP were treated with ceftriaxone-monotherapy (16/69, 23.2%), penicillin-G (12/69, 17.4%), or ampicillin and gentamicin (11/69, 15.9%) as recommended by the WHO: using ampicillin (or penicillin) with gentamicin as first-line or ceftriaxone as second-line for treatment of severe CAP among children aged 2 to 59 months. Other antibiotic combinations used included penicillin-G and chloramphenicol (6/69, 8.7%), ceftriaxone and cloxacillin (4/69, 5.8%), and ampicillin and cloxacillin (2/69, 2.8%). Ampicillin was dosed at 50 mg/kg for eight patients, one patient received a lower dose; penicillin-G was dosed at 50,000 units/kg for 19 patients, two patients received a higher dosage. There was no use of macrolides, tetracyclines or fluoroquinolones.

Forty-eight (68.6%) of the patients with sepsis (70) had cultures requested, of which four (8.3%) had bacteria isolated and respectively treated with ceftriaxone and ciprofloxacin (*Acinetobacter baumanii,* from cerebrospinal fluid, with no reported resistance); ampicillin and gentamicin combination, which were changed to ciprofloxacin (C*oliform species*, from oral-swab, with resistance reported to ampicillin and gentamicin); ampicillin and gentamicin combination, which were changed to ceftriaxone, and later to ciprofloxacin (*Salmonella species*, from stool, with resistance reported to ampicillin); ceftriaxone and cloxacillin (*Staphylococcus aureus*, from blood, with no reported resistance). Nine (81.8%) of the patients with UTI (11) had cultures requested, of which two (22.2%) had bacteria isolated from urine and were treated with ampicillin and gentamicin combination, later changed to ceftriaxone, with additions of nitrofurantoin and ciprofloxacin during the course of treatment (*Escherichia coli and Klebsiella species*, with resistance reported to ampicillin for both organisms); ampicillin and gentamicin combination (*Coliform species*, with resistance reported to ampicillin). Thus all the resistant bacteria reported (five) were to ampicillin (5/5, 100%) and gentamicin (1/5, 20%). The difference in the choice of antibiotics between these patients with or without positive cultures was not significant.

### Bacterial cultures and antibiotic resistance testing

**Fig. 2:**
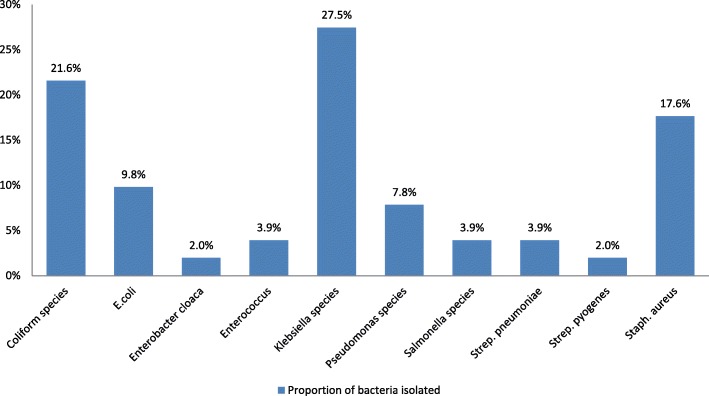
Laboratory data on isolated bacteria from microbiological cultures of patient specimens from January–December 2015

At least one culture request was indicated in the admissions records for 266/496, 53.6% of the patients who were on antibiotics. There were 51 clinically relevant bacterial isolates from the laboratory records. The most common were *Klebsiella species* (14/51, 27.5%, from urine 13/14, 92.9% and aspirate 1/14, 7.1%), *Coliform species* (11/51, 21.6%, from urine 7/11, 63.6%, swabs 2/11, 18.2%, and for each specimen (sputum and aspirate) 1/11, 9.1%), and *S. aureus* (9/51, 17.6%, from swabs 7/9, 77.8%, and for each specimen (blood and aspirate) 1/9, 11.1%) (Fig. 2).

**Fig. 3:**
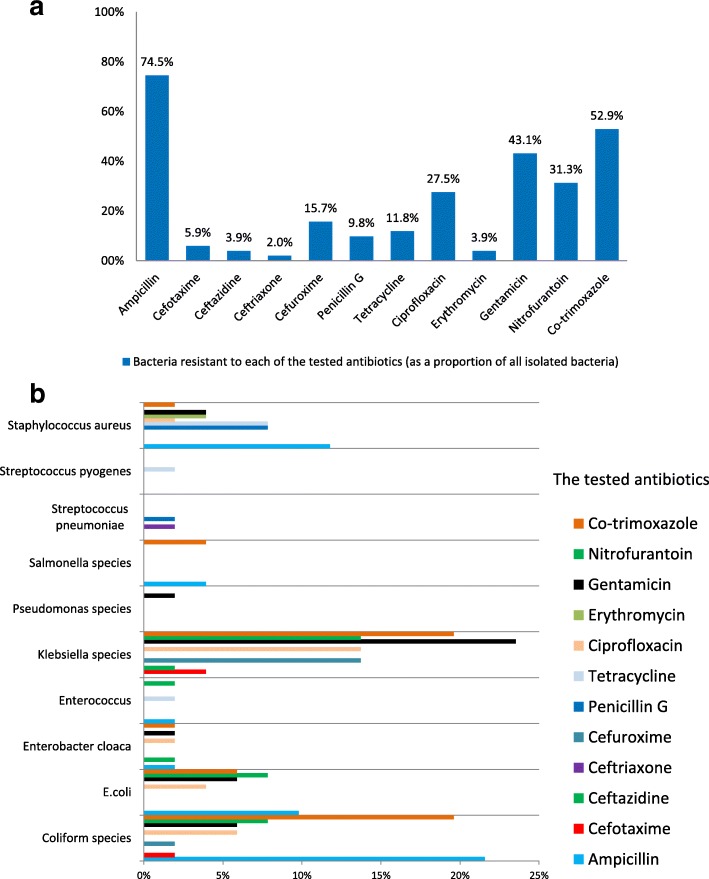
**a** Laboratory data on antibiotic resistance testing from January–December 2015. **b** The distribution of the reported antibiotic resistant bacteria to all the tested antibiotics

Antibiotic resistance was mainly to ampicillin (38/51, 74.5%, mainly reported as *Coliform species* 11/51, 21.6%, *S. aureus* 6/51, 11.8%, and *E. coli* 5/51, 9.8%. The proportion of the reported resistance of *Klebsiella species* to ampicillin was excluded due to its intrinsic resistance to ampicillin), co-trimoxazole (27/51, 52.9%, mainly reported as *Coliform species* and *Klebsiella species* 10/51, 19.6% for each organism, *E. coli* 3/51, 5.9%, and *Salmonella species* 2/51, 3.9%), and gentamicin (22/51, 43.1%, mainly reported as *Klebsiella species* 12/51, 23.5%, for each organism (*Coliform species* and *E. coli*) 3/51, 5.9%, and *S. aureus* 2/51, 3.9%) (Fig. 3). Fig. 3b illustrates the reported antibiotic resistant bacteria to all the tested antibiotics. Antibiotic resistance to third generation cephalosporin was 6/51, 11.7%, all were Enterobacteriaceae thus suggestive of extended spectrum beta lactamase producing (ESBL), isolated from urine (3/6, 50%) and for each specimen (blood, aspirate, and swabs) 1/6, 16.7%.

## Discussion

Our retrospective study conducted amongst paediatric inpatients in the highest referral hospital in the Gambia shows more than half of the admitted patients received at least one antibiotic and slightly more than half of these patients had microbiological cultures indicated. Antibiotic resistance was high for the most commonly used antibiotics (ampicillin and gentamicin).

We observed an overall higher antibiotic consumption (670.7 DoT/1000 PD) when compared to that observed from a pre-interventional phase of an ABS study conducted in a paediatric unit in a developed country (483.6 DoT/1000 PD) [19]. The volume of antibiotic used in our study was higher for all comparable antibiotics except for metronidazole, ciprofloxacilln and vancomycin. The proportion of patients treated with antibiotics was as similarly observed at the health-center outpatient level among children under 5 years old (63.4%), and higher among admitted neonates (94%) in the Gambia [11, 17]. In other developing countries, a similar proportion of children received antibiotics during admission, ranging from 63.6% in Indonesia to 71.1% in Nigeria [3, 21]. The wide difference in the use of antibiotics between developing and developed countries could be related to multiple factors such as the higher rate of infectious diseases in developing countries [3, 29], the limited access to diagnostic parameters to confirm definitive need for antibiotic use, the limited access to support from specialists such as infectious disease specialists, and lack of local antibiotic policies in developing countries [1, 30]. From our study, the availability of microbiology results was useful in guiding the selection of the right antibiotic class as demonstrated in the treatment of sepsis and UTI, although the limited available results may have affected the statistical significance.

Empirical antibiotic indication and dosage for the treatment of severe CAP were as recommended by WHO for most of the patients [24]. A smaller proportion of patients were treated with penicillin-G and chloramphenicol combination although this has been reported in the WHO recommendation as inferior to ampicillin and gentamicin combination for treating severe CAP. This may be partly explained by availability of penicillin and chloramphenicol. The other antibiotics used for few of the patients are however not in the WHO recommendations, possibly based on specific clinical judgement such as failure of first and second-line therapies started from a referral hospital, high clinical suspicion for a specific organism based on the clinical presentation, or drug availability. The disruption of drug supply in developing countries affecting drug availability and appropriate antibiotic use has been reported [7, 31]. Ceftriaxone, which belongs to the WHO WATCH group of antibiotics often used as second-line treatment [32] was one of the most common antibiotics used, with a higher density of use than ampicillin which is a first line drug. Although at our study site the prescription of this drug is controlled, as it has to be countersigned by a specialist, the limited availability of other second-line drugs may have contributed to its frequent use.

About half of the patients treated with antibiotics had at least one microbiology test requested. A high proportion of empirical antibiotic use based on clinical judgement was also reported in The Gambia among neonatal admissions [17], and elsewhere in Africa [29]. This limited microbiological laboratory use in developing countries may also reflect a lack of trust into the value of microbiological results, possibly due to the limited laboratory services, delays in the provision of results, amongst others [33, 34]. Clinical suspicion of co-infections or severe infections such septicemia may have also warranted the immediate administration of relevant antibiotics, pending later microbiological investigation.

Although the species for many of the organisms were not identified, the bacteria belong to the groups of the most dangerous resistant pathogens including both gram positive and negative organisms as reported by Fair et al. [35]. They also reported stability in the resistance rates of gram positive organisms, on the contrary, gram negative organisms’ resistance rates tend to be on the rise. This finding is similar to our results, as most of the reported resistant pathogens were gram negatives and multi-drug resistant as defined by Magiorakos et al. [36]. The more difficulty in treating gram negatives could be explained by their resistant mechanisms especially their added mechanism of inhibiting antibiotic penetration due to the existence of an outer membrane, and the higher presence of efflux pumps over-expression compared to gram positives, in the presence of the other possible mechanisms [18, 35]. The recorded resistance towards the commonly used antibiotics (ampicillin and gentamicin), and considering that some of the isolates are suggestive of ESBL Enterobacteriaceae which is one of the 12 important bacteria families highlighted by the WHO needing more attention [37], conducting microbiological workup prior to the onset of empirical treatment could encourage a more rational antibiotic use and limit resistance. A review on antibiotic resistance in Africa also reported a high bacterial resistance to the first line antibiotic therapy and Enterobacteriaceae to third generation cephalosporin [38]. Although use of co-trimoxazole in our study was low, the resistance reported was among the highest. This could be explained by the high use of this antibiotic in the outpatient health centers where availability of the other antibiotics maybe limited [11].

### Limitations

The possibility of missing data from the admission records especially the microbiology data makes it difficult to make absolute judgements on the use of antibiotics based on laboratory findings. The data we obtained from the laboratory could not resolve this problem because some of the records lack hospital numbers thus matching this data with the admission data was not feasible. Many of the organisms were reported as genus without identification to the species level, making it difficult to compare with other settings and specify the resistant organism more appropriately.

## Conclusion

Our study shows that more than half of the admitted patients received antibiotics although most of the available results showed no microbiological evidence for their indication, suggesting that most of the antibiotics were empirically prescribed. Although several other factors in such settings could contribute to use of antibiotics for patients without microbiological evidence, the availability of microbiology results was a useful guide for choosing the class of antibiotic. Thus, in addition to the use of standards to guide empirical antibiotic therapy such as that of the WHO, microbiological use to guide antibiotic prescribing should be encouraged. This is probably achievable through better access to laboratory services and the establishment of ABS and its promotion for acceptance. Our study has shown high bacterial resistance to commonly used antibiotics, including ESBL bacteria, warranting the need for further research on the local antibiotic resistance patterns of bacteria as well as setting up an antibiotic resistance surveillance system.
